# Evaluation of Hospice@Home for Home-Based Palliative Care: Development and Usability Pilot Study

**DOI:** 10.2196/79334

**Published:** 2026-01-21

**Authors:** So-Hi Kwon, Mikyoung Angela Lee, Young-Joo Kim, Seo-Hye Park, Min-Jeong Lee, Youngmin Han, A-Sol Kim

**Affiliations:** 1 Research Institute of Nursing Innovation, College of Nursing, Kyungpook National University, 80, Daehak-ro, Buk-gu, Daegu, 41566, Republic of Korea, 82 10-8564-5345; 2 College of Nursing, Texas Woman's University, Dallas, TX, United States; 3 Hospice Palliative Care Center, Kyungpook National University Chilgok Hospital, Daegu, Republic of Korea; 4 School of Smart Industry, Kyungil University, Gyeongsan, Republic of Korea; 5 Department of Family Medicine, School of Medicine, Kyungpook National University, Daegu, Republic of Korea; 6 Kyungpook National University Chilgok Hospital, Daegu, Republic of Korea

**Keywords:** digital health, home care services, hospice care, usability, wearable devices

## Abstract

**Background:**

The demand for palliative care is rising due to population aging and increased chronic illness. However, access to timely palliative care remains limited, particularly for patients receiving home-based hospice care in rural areas. Digital health technologies present an opportunity to enhance care delivery and communication at home.

**Objective:**

This pilot study aimed to (1) develop the Hospice@Home system, a digital in-home hospice care solution; (2) explore preliminary indications of usability and feasibility among patients with terminal cancer and their caregivers; and (3) identify challenges for future implementation.

**Methods:**

Hospice@Home was developed following the human-centered, evidence-driven Adaptive Health Experience and Application Design approach. After the prototype was developed, alpha testing was conducted with 2 simulated patients to assess system functionality and identify technical issues. Usability was measured through structured observation and task completion success during these sessions. Feasibility was evaluated during a 3-week beta test involving 5 dyads of patients with terminal cancer and caregiver, recruited through a home-based palliative care agency. Challenges were identified through user feedback, field notes, and technical logs collected throughout the testing period.

**Results:**

Hospice@Home is a web app optimized for Android devices. It integrates wearable biosignal data—blood pressure, pulse rate, sleep patterns, and oxygen saturation—measured via the Samsung Galaxy Watch 6. It also allows self-reporting of body temperature, pain levels, bowel movements, and the severity of symptoms tailored to individual patients. Medication compliance, including scheduled and pro re nata analgesics, was recorded in a smart medication box and automatically transmitted to Hospice@Home. Over 3 weeks, 5 patients (aged 53‐93 y) with terminal cancer and their caregivers (aged 38‐63 y) explored the system. Both patients and caregivers appreciated the consolidated symptom reporting and real-time data sharing, noting that the system helped them feel more reassured and connected to clinical support. Usability was assessed via satisfaction ratings, averaging 3.3 (SD 0.5) for patients and 4.0 (SD 0.7) for caregivers (5-point scale). Feasibility was evaluated through task compliance; dyads completed ≥13 of 18 tasks during stable periods. No major technical issues were reported. Challenges to consistent system use included data entry fatigue, psychological barriers to using technology, fluctuations in cognitive and physical functioning, and a general preference among patients and caregivers for phone calls rather than using the in-app communication.

**Conclusions:**

Hospice@Home showed early signals of usability and feasibility among patients with terminal cancer and caregivers, but these preliminary observations require cautious interpretation given the pilot design. The findings highlight potential value in supporting home-based palliative care, while underscoring the need for ongoing refinement and iterative testing before making broader claims about effectiveness or clinical impact.

## Introduction

Palliative care is a specialized approach for people with serious, chronic, or life-threatening illnesses and has demonstrated positive outcomes, including improved quality of life, reduced health care costs, and high satisfaction among patients, families, and care providers [1]. The global demand for palliative care has been steadily increasing, driven by an aging population and rising prevalence of chronic diseases [2]. Having become a super-aged society in 2024 [3], South Korea is preparing for the implementation of the Act on Integrated Support for Local Medical and Long-Term Care Services in 2026, which is designed to strengthen community-based care that supports individuals through the end of life [4]. In South Korea, palliative care is provided through 3 main models: palliative care team, palliative care unit (PCU), and home-based palliative care (HPC) [5]. Palliative care team services are offered in general wards in hospitals or cancer centers, while PCU care is provided in dedicated palliative care wards within hospitals. HPC is delivered by dedicated home hospice nurses in the interdisciplinary PCU team, allowing for continued care in patients’ homes. Within this system, national policy efforts have focused on expanding the availability and accessibility of home-based palliative care services.

For patients with cancer, HPC offers significant benefits over nonpalliative care, including higher patient satisfaction, fewer emergency room visits, and significantly lower medical costs [6]. Additionally, it has been reported to be more cost-effective than the care provided in PCUs [7]. Despite these advantages and growing demand, HPC remains underutilized in South Korea. While there are 92 PCUs, only 39 institutions offer HPC, serving just 3.5% of the hospice users nationwide [5]. Most palliative care in South Korea is delivered in acute care settings within general hospitals or higher-level facilities, primarily concentrated in urban areas [3]. Access to HPC in rural regions remains limited due to workforce shortages, including a lack of adequately trained personnel, resulting in disparities in care [8]. Paradoxically, rural areas with a higher proportion of older adults and greater need for palliative care often have fewer HPC services available, highlighting a mismatch between supply and demand. To mitigate this imbalance, strategic approaches are needed to strengthen service delivery capacity and ensure equitable access to HPC across regions.

In home settings, patients and caregivers often face challenges in managing symptoms and experience significant caregiving burdens, which may contribute to the low utilization of HPC [9]. Therefore, it is essential to improve the home care environment by developing efficient management systems that can maintain consistent quality of care even with limited human resources. Effective symptom management in HPC depends on precise monitoring, clear communication among patients and health care providers, and timely intervention. However, unlike inpatient settings, HPC lacks continuous 24-hour monitoring and often relies on patients’ recall rather than real-time symptom reporting or objective data, such as vital signs. As a result, decision-making for symptom management, including medication adjustments, is frequently delayed until a nurse conducts an in-person visit. These delays often lead patients to prefer PCU admission. When PCU bed availability is limited or geographic access is difficult, patients and their families often opt for admission to nursing homes or geriatric hospitals as an alternative. In either case, patients are ultimately institutionalized, unable to remain at home during their final stage of life. The integration of digital technologies into home care can address these challenges by improving efficiency, accessibility, and the overall quality of care.

Digital health (DH) technologies—including wearables, telehealth, and mobile apps—offer promising opportunities to improve HPC by enabling remote symptom monitoring, tracking treatment adherence, and supporting decision-making. For example, Huang et al. [10] demonstrated that activity metrics collected through wearable devices could be used to assess patients’ functional status and predict prognosis in hospice care settings, while Phongtankuel et al [11] reported the potential of mobile health apps to facilitate communication and symptom tracking in home hospice care. Beyond palliative care, remote patient monitoring has also shown measurable benefits in other clinical contexts, such as reduced hospital length of stay and improved care coordination among patients with COVID-19 [12]. These technologies may help overcome geographic barriers and improve continuity of care. However, several concerns have been raised regarding the feasibility and ethical implications of using digital technologies in end-of-life contexts, including patient fatigue, technology anxiety, data privacy issues, and the risk of depersonalizing care [11,13].

Evidence from recent reviews suggests that DH interventions in palliative care produce encouraging yet mixed results, showing promising effects, such as improved symptom management, communication, and caregiver support, but also notable methodological limitations, including small sample sizes and a lack of methodological rigor as clinical studies, with most research focusing on patients in earlier palliative stages rather than those receiving end-of-life care at home [14,15]. In addition, most systems applied fixed, one-size-fits-all symptom assessment tools that increased patient burden and did not account for individual variability in symptoms. Integration with real-time biosignal monitoring or existing electronic medical records was also largely absent, hindering clinical applicability. To address these gaps, the development of DH systems capable of timely symptom recognition, medication tracking, and effective communication tools within home settings has been recommended. Yet, despite growing global interest, the application of DH technologies in HPC remains largely unexplored in Southeast Asia, including South Korea [16]. Recent consensus efforts and policy discussions have emphasized the need to establish standardized evaluation frameworks, ensure interoperability with existing health systems, and expand reimbursement pathways to accelerate the adoption of DH in palliative care [17,18]. However, despite these global efforts, research specifically examining the usability and feasibility of DH in HPC in South Korea remains scarce. Most existing studies have primarily focused on DH interventions for relatively stable patients or those with chronic diseases, rather than for individuals at the end of life, and few have conducted empirical evaluations of tailored systems in HPC settings. Assessing the practical feasibility and user experience of such a system in this context is therefore essential to guide further development and large-scale implementation. This gap provides the rationale for this pilot study.

In response to these challenges, this study developed Hospice@Home*,* a digital home−based palliative care system designed to enhance the delivery of palliative care for patients in their homes. The system integrates remote biosignal monitoring, electronic medication tracking, and a patient-reported symptom management interface to support timely and personalized care. The aim of this pilot study was to develop Hospice@Home and evaluate its feasibility and usability. The research questions were as follows:

1. Is the Hospice@Home system usable and feasible in home-based palliative care?
2. What challenges and contextual factors affect its implementation?

## Methods

### Ethical Considerations

This study was approved by the Kyungpook National University Hospital Institutional Review Board (KNUH IRB 2023-12-045-002), and written informed consent was obtained from all patients and their family caregivers before participation in the study. Participant privacy was protected, and all collected data were anonymized and kept confidential. Participants received an in-kind compensation in the form of a thermometer valued at 100,000 KRW (approximately US $68) for their participation.

### Study Design

This pilot study employed a mixed methods approach combining quantitative data collection and qualitative interviews with patients and caregivers. This study was guided by the Approach to Human-centered, Evidence-driven Adaptive Design (AHEAD), a systematic methodology that integrates empathy-driven problem-solving approach with the core stages of inspiration, ideation, and implementation [19]. The AHEAD framework involved five key steps: (1) defining the problem and assembling a team, (2) information gathering, (3) synthesis, (4) intervention design, and (5) evaluation. These steps were executed collaboratively by an interdisciplinary team comprising experts in palliative care, home care, engineering, and software development.

### System Development

#### Step 1. Define the Problem and Assemble a Team

An interdisciplinary subject matter experts (SME) team was assembled, comprising a palliative care physician with 10 years of experience, a home hospice nurse from a regional hospice center, and 2 hospice advanced practice nurses. This team collaboratively identified key weaknesses in current HPC practices compared to PCU, including:

- Inconsistent monitoring of vital signs and symptoms
- Medication management challenges, such as missed fentanyl patch replacements and inaccurate pain logs, hindering pro re nata (PRN, as needed) medication tracking
- Limited provider-patient communication between visits due to connectivity constraints
- Delayed provider response to changes in patient condition due to insufficient real-time objective data

Based on these gaps, the SME team established core priorities for high-quality HPC. These included timely symptom and vital sign monitoring, accurate and proactive medication documentation and management, responsive provider-patient communication between home visits, and integration of real-time patient–reported outcomes to support clinical decisions.

#### Steps 2 and 3. Information Gathering and Synthesis

To address these challenges, the SME team conducted reviews of literature and online resources, consulted with DH companies, and piloted potential products. This process led to consensus on the essential features of the Hospice@Home system:

##### Real-Time Monitoring of Vital Signs and Symptoms and Data Integration

For the continuous, centralized tracking of vital signs and symptoms, the SME team evaluated wearable devices for their compatibility, reliability, and capacity to collect key biosignal data. The Galaxy Watch 6 was selected for its compatibility with Android platforms and ability to monitor key indicators, such as blood pressure (BP), pulse rate (PR), oxygen saturation (O_₂_Sat), and sleep patterns. These biosignal parameters offer critical insights into patients’ health status. For symptom assessment, the team reviewed validated instruments such as the Palliative Outcome Scale, Symptom Assessment Scale by Palliative Care Outcomes Collaboration, and the Edmonton Symptom Assessment Scale [20]. Most standardized symptom assessment tools for terminally ill patients evaluate 7‐9 common symptoms (eg, pain, fatigue, nausea, depression) using Likert scales. These tools also include open-ended items that allow patients to report and rate additional symptoms not listed. However, requiring patients to score every symptom—regardless of personal relevance—can increase reporting burden. To reduce reporting burden and improve compliance, the SME team recommended limiting assessments to 3 target symptoms—including pain—tailored to each patient’s care needs. The Bristol stool chart was also adopted to standardize bowel diary entries. The system design incorporated functionality allowing patients and caregivers to transmit symptoms and biosignal data directly to providers, enabling remote, real-time monitoring and proactive intervention.

##### Smart and Flexible Medication Management

The SME team conducted a comparative analysis of commercially available smart medication boxes (SMBs), assessing features, such as reminder settings, support for various dosage forms and PRN medication, portability, ease of use at home, and affordability [21,22]. Most market-available SMBs, including pill organizers, vial caps, and alarm-enabled dispensers, were found to primarily accommodate solid-form medications, making them unsuitable for patients with palliative care who often require multiple medication forms (eg, oral solutions, transdermal patches, nasal sprays) and the complexity of palliative medication schedules (eg, opioid patch replacements every 48‐72 h or oral PRNs taken based on symptom intensity). Given these limitations, the team opted to develop a customized SMB prototype capable of supporting multiple formulations and flexible dosing intervals, along with built-in reminder functions and adherence alerts tailored to each patient’s regimen.

##### Responsive Provider-Patient Communication

Limited communication between home visits was identified as a major gap in care continuity. To address this, the SME team emphasized the need for real-time communication pathways. Therefore, when patients press the emergency button, send a message, or when predefined thresholds are exceeded, automated alerts are immediately sent to health care providers. The system interface—customized for administrators, providers, and patients, respectively—displays structured visualizations of symptoms, vital signs, and medication adherence to support timely interventions and communication.

##### Personalized Alerts for Early Intervention

Delays in clinical decision-making were often attributed to the lack of timely and actionable data. To overcome this issue, the system was designed to incorporate automated alerts triggered when biosignal values or symptom severity deviate from predefined parameters. These real-time notifications enable providers to intervene earlier, preventing symptom escalation and unnecessary hospital visits.

##### User-Friendly Design

The interface featured large, high-contrast fonts and simple icons, with 1‐2 step navigation to reduce cognitive load. Interactive elements, such as symptom sliders and medication checkboxes, allowed easy touch input. Text-to-speech system supported users with low literacy. These features aimed to accommodate older adults and cognitively or physically vulnerable users.

##### System Architecture and Security

**Figure 1.**
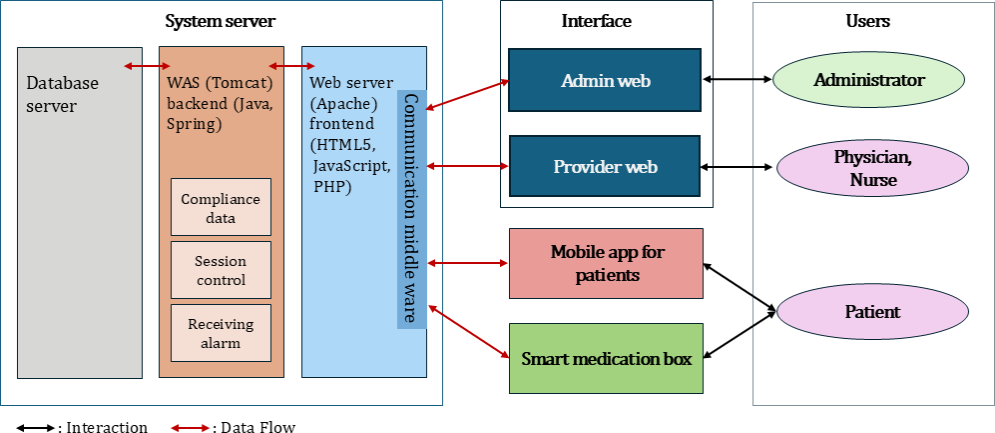
Hospice@Home system structure. WAS: web application server.

The Hospice@Home System was built on Naver’s Linux server infrastructure, incorporating a dedicated database server for secure data storage and a web app server for managing compliance data, session control, and alerts using Java Spring Legacy (Figure 1). Two web-based interfaces—one for administrators and another for health care providers—were developed using HTML5 and JavaScript to support role-specific functionality. The provider web interface also integrated a specialized alarm feature, developed in Flutter, that enabled nurses to receive and confirm alerts triggered by patient-reported symptoms and biosignal thresholds. This functionality allowed nurses to monitor patients in real time and respond promptly, even outside scheduled visits. The patient-facing Android mobile app, also built with Flutter, supported notifications and interactive features.

Secure and standardized data exchange was enabled through HL7 Fast Healthcare Interoperability Resources [23], while health care data and activity logs were managed by the Tibero 7 database management system [24], utilizing role-based access control to maintain data integrity and privacy. These components ensured reliable integration and secure transmission of medication usage data from the SMB, including compliance tracking, missed dose alerts, and timestamped medication logs. This supported real-time monitoring of medication adherence and facilitated clinical decision-making based on objective records. To safeguard personal information, the system implemented bidirectional encryption for personal data and unidirectional encryption for passwords, in full compliance with the Personal Information Protection Act [25]. A minimum viable product of the Hospice@Home system was developed and underwent multiple rounds of testing and refinement. The iterative process focused on optimizing system performance, enhancing user experience, and addressing potential issues to ensure reliable functionality.

### Step 4. Intervention Design

**Figure 2.**
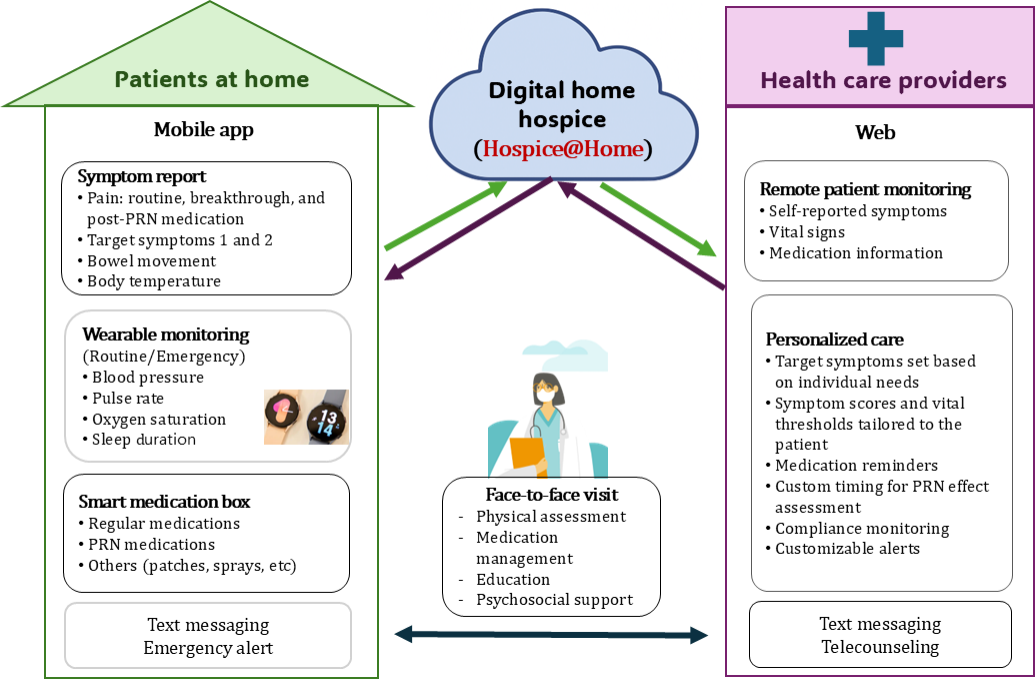
Hospice@Home service framework. PRN: pro re nata.

In this step, the AHEAD framework emphasized establishing guiding principles and exploring ideation [19]. Accordingly, our study team developed the Hospice@Home service framework, outlining the workflow and information flow of this service (Figure 2). The Hospice@Home service framework facilitates coordinated palliative care for patients with terminal illnesses at home. The system integrates patient self-reporting, wearable biosignal monitoring, and smart medication tracking through a mobile app. Each day, patients or caregivers enter symptom data (eg, pain, bowel movement, body temperature) and confirm medication intake, while wearable devices automatically transmit vital signs, including BP, PR, O_2_Sat, and sleep duration. All data are sent to the cloud-based platform in real time. Health care providers access this information through a web interface, which displays structured dashboards of patient-reported outcomes, vital signs, and medication adherence. The system is designed to track PRN medication use alongside pain scores, enabling clinicians to monitor the effectiveness of analgesics and detect patterns of breakthrough pain. Based on the patient’s condition, providers set personalized symptom thresholds and medication schedules. When data exceed these thresholds—or when patients activate the emergency button or send a message—automated alerts are triggered, prompting timely clinical response. Providers may send instructions or reminders via in-app messaging or initiate teleconsultation as needed. Face-to-face visits supplement digital care by addressing complex needs such as physical assessment, medication titration, education, and psychosocial support. This hybrid approach ensures that communication and clinical decision-making remain responsive and patient-centered throughout the course of home-based hospice care.

To evaluate the functionality, the Hospice@Home prototype, which included the Galaxy Watch6, SMB, and Hospice@Home app, we conducted alpha testing using simulated scenarios with virtual patients and prescriptions. Two nurses alternated as simulated patients to identify potential usability issues and system malfunctions. During testing, the following issues were detected and addressed through system updates: (1) unstable Wi-Fi connectivity—addressed by implementing mobile hotspots to ensure consistent network access, (2) missed app alerts caused by delayed SMB synchronization—resolved by optimizing the communication interval between the app and SMB and reducing latency through firmware updates, (3) intermittent errors in biosignal display on the Galaxy Watch—corrected by updating to the latest wearable OS, and (4) incorrect fentanyl patch scheduling due to input errors in the initial dosing time—mitigated through a built-in reminder function for patch replacement. Additionally, a detailed user manual was created in both a printed booklet and a series of short video clips to guide users in navigating system functionalities and troubleshooting potential issues.

### Usability and Feasibility Testing

Usability and feasibility testing were conducted as part of Step 5 (Evaluation) of the AHEAD framework.

#### Participant Recruitment

Participants were recruited consecutively from a single home–based palliative care center over a 6-month period (February-October 2024). Eligible participants were newly enrolled in HPC and met the following inclusion criteria: (1) ability to report symptoms using numeric rating scale, (2) access to wireless internet, and (3) willingness to provide informed consent. Recruitment leaflets and advertisements were posted in a PCU. Initial screening was conducted by the attending physician to confirm cognitive capacity for participation. Subsequently, a home hospice nurse conducted an in-home assessment to evaluate the internet environment and caregiver availability. Patients meeting these criteria were introduced to the study and, upon agreement, received a visit from the researcher, who provided system training and obtained written informed consent.

During the study period, 7 patients were recommended by the attending physician and subsequently screened by a home hospice nurse to verify eligibility based on home internet connectivity and caregiver support. Of these, 1 patient declined participation after the researcher’s explanation due to discomfort with technology, and another withdrew during the study because of rapid clinical deterioration. Ultimately, 5 (71%) patient-caregiver dyads consented to participate and completed the beta testing. Although the final sample size was small, this is consistent with recommendations for pilot studies that aim to gain meaningful insights into the usability of a system rather than statistical generalizations [26].

#### Training

Each participant was provided with a Galaxy Watch 6, a Galaxy A13 smartphone preloaded with the Hospice@Home app, and an SMB. Patients and caregivers received hands-on training using a structured user manual. Additionally, a structured 2-hour training workshop was held for the attending physician and hospice nurses. The training covered key system functionalities, including user login, prescription entry, patient data review, and use of in-app chat feature. To enhance mobility and responsiveness, the app was also installed on nurses’ personal smartphones, enabling real-time notifications and patient monitoring in the field.

#### Feasibility Measures

Feasibility testing was conducted over a period of 3 weeks. This duration was determined with reference to national hospice data in South Korea, where the average hospice enrollment lasts approximately 27 days [5]. Although the average survival of home hospice patients is around 2 months, a 3-week period was chosen to balance the need for sufficient observation with minimizing burden on patients with terminal illnesses and caregivers.

In this study, feasibility was defined as the extent to which the Hospice@Home system could be implemented and used as intended in HPC environment. Feasibility testing focused on two core domains: (1) technical feasibility, evaluated by examining the reliability of biosignal measurements obtained through the system, and (2) user feasibility, assessed through patients’ and caregivers’ engagement with symptom reporting and medication compliance. The reliability of biosignal measurements was assessed by comparing Galaxy Watch 6 data with readings from standard reference devices during the first 3 nurse home visits, each conducted on a separate day. At each visit, a single comparison was made for systolic and diastolic BP, PR, and O_₂_Sat, using the Omron HEM-7121J and Dr. Healing HPO-010 as reference devices. A total of 60 paired measurements (systolic blood pressure, diastolic blood pressure, PR, and O_₂_Sat), measured across 3 separate visits in 5 participants, were collected for intraclass correlation coefficient (ICC) analysis. Sleep duration was excluded from the ICC analysis due to difficulty in distinguishing sleep from rest periods and the lack of consistent verbal reports from patients, which limited comparison with reference data.

Symptom reporting and medication compliance were analyzed through system-generated compliance metrics extracted from the administrator dashboard. These metrics included vital sign measurement, symptom reporting, and medication use pattern. Compliance was scored as follows:

1. Vital sign compliance: Each of the 4 daily vital sign measurements (BP, PR, body temperature, O_₂_Sat) was scored separately, with 2 points for full completion, 1 point for at least 1 measurement, and 0 points for none, for a total possible score ranging from 0 to 8 points.
2. Symptom reporting: 2 points were assigned for daily entries including pain and at least 2 additional symptoms (eg, dyspnea), and 0 points for no entry, yielding a daily score ranging from 0 to 6.
3. PRN pain score reporting: 2 points for both pre- and postmedication pain reports, 1 point for prereporting only, and 0 points for no report. If no PRN medication was administered during the evaluation period, the case was scored as 2 points, indicating full adherence.
4. Scheduled medication use pattern: 2 points for full completion of regularly scheduled medications (excluding PRN use) and 0 points for any omissions.

The total possible compliance score ranged from 0 to 18 points, reflecting overall engagement with the system and adherence to the clinical protocols.

#### Usability Measures

The 5-item usability questionnaire used in this study was developed by the research team to reflect core aspects of user experience, including overall satisfaction, ease of use, perceived helpfulness, likelihood of recommendation, and communication improvement. These domains represent the key constructs commonly assessed across validated usability instruments such as the System Usability Scale, the mHealth App Usability Questionnaire, and the Mobile Application Rating Scale [27]. Given the frailty of the target population, a brief 5-item format was adopted to reduce respondent burden. Although this simplified tool was not formally validated, it was deemed acceptable for this early-stage feasibility study, and the use of validated instruments is planned for future large-scale evaluations.

Qualitative feedback on system features was also gathered to supplement usability ratings and provide contextual insight into caregivers’ experiences with system use. Although all 5 patient-caregiver dyads who completed the beta testing were eligible for qualitative interviews, the patients were unable to participate due to clinical deterioration. Consequently, qualitative interviews were conducted with all 5 caregivers. To collect these qualitative data, caregivers participated in in-home interviews conducted at least once per week over the 3-week evaluation period. Before each interview, written informed consent for audio recording was obtained. Interviews were held in the patient’s home, incorporated brief field observations, lasted approximately 60 minutes, and were audio-recorded and transcribed verbatim. These interviews were conducted throughout the 3-week evaluation period and were used to supplement usability ratings by providing deeper insight into caregivers’ experiences, including challenges encountered during use, features they found helpful, and suggestions for improvement.

### Statistical Analysis

Statistical analysis was performed using Microsoft Excel and IBM SPSS Statistics (version 28). Descriptive statistics, including means, SDs, frequencies, and percentages, were used to summarize participants’ demographics, compliance metrics, and user satisfaction scores. To evaluate the reliability of biosignal measurements obtained from the Galaxy Watch 6 in comparison to standard reference devices, ICCs and 95% CIs were calculated using a 2-way random-effects model for absolute agreement based on average measures. This model was selected because the wearable device and reference device represent random samples of potential measurement tools, and the aim was to assess absolute agreement rather than consistency. ICC values were interpreted using commonly applied thresholds (<0.50 = poor, 0.50‐0.75=moderate, 0.75‐0.90=good, and >0.90 = excellent agreement). Quantitative results were then integrated with qualitative findings to provide contextual explanations of usability scores and compliance patterns, following a complementary mixed methods framework. Specifically, qualitative themes related to usability barriers (eg, fatigue, cognitive fluctuations) and facilitators (eg, reassurance, data consolidation) were compared with individual-level compliance trends and satisfaction ratings. This comparison allowed us to identify converging and diverging patterns across qualitative experiences and quantitative engagement metrics. Together, these mixed methods findings provided contextual explanations for variability in engagement and supported interpretation of the system’s preliminary performance.

### Qualitative Analysis

Qualitative data from patient and caregiver interviews, along with researchers’ field notes, were analyzed using conventional content analysis [28]. This approach was chosen to allow categories and themes to emerge inductively from the data without imposing preexisting theoretical frameworks. Two researchers independently coded interview transcripts and field notes, identified recurring meaning units, and discussed discrepancies until agreement was reached. Coding proceeded through 3 stages: initial open coding, grouping of codes into broader conceptual categories, and iterative development of themes. A shared codebook was developed and refined throughout the analysis, and discrepancies between coders were discussed until consensus was achieved. Themes were organized to capture shared and divergent experiences among patients, caregivers, and health care providers, and the codebook was refined iteratively through discussion. Although the small pilot sample did not allow for full data saturation, the thematic patterns provided preliminary insights into system usability, engagement barriers, and implementation considerations in home hospice care.

## Results

### Interfaces of the Hospice@Home App

**Figure 3.**
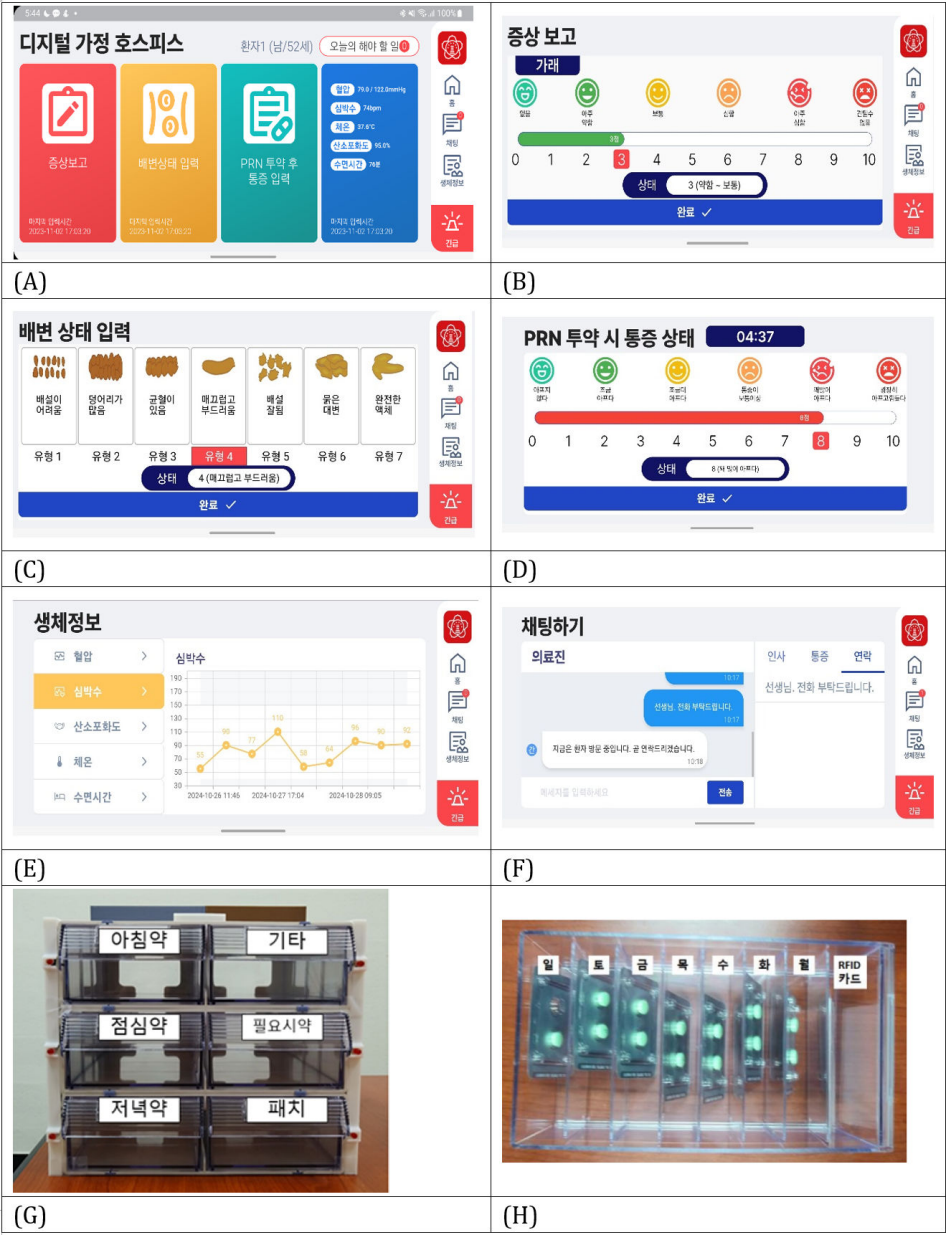
Patient-facing mobile app interface and smart medication box. (A) Main screen. (B) Symptom report. (C) Bristol constipation scale. (D) Pain score at the time of taking pro re nata (PRN) medication. (E) History of vital signs. (F) Chat with provider. (G) Front view of the smart medication box. (H) Inside of the smart medication box.

The Hospice@Home mobile app allowed patients to record daily information including pain intensity (both routine and post-PRN), bowel movements, target symptoms, and medication intake times. The app also provided a view of vital sign trends collected via the Galaxy Watch6, a chat function for asynchronous communication with providers, and an emergency button to request immediate assistance (Figure 3A-F). The patient-entered data were transmitted in real time to the provider’s system to support continuous monitoring.

Integrated via Wi-Fi, the SMB supported scheduled and PRN medication adherence through a drawer-type radio frequency identification (RFID)–tagged system. Voice and light alerts reminded patients of scheduled doses, while RFID tagging verified actual medication removal—unlike conventional devices that only detect box opening. For PRN analgesics, tagging prompted the app to request a predose pain rating and later sent a reminder for postdose reassessment. Real-time intake data were automatically transmitted to the mobile app, provider interface, and server. The SMB accommodated over 7 days of medications, including oral drugs, opioid patches, and PRNs, and was designed with LED indicators and voice guidance tailored to older adult users (Figure 3G,H).

**Figure 4.**
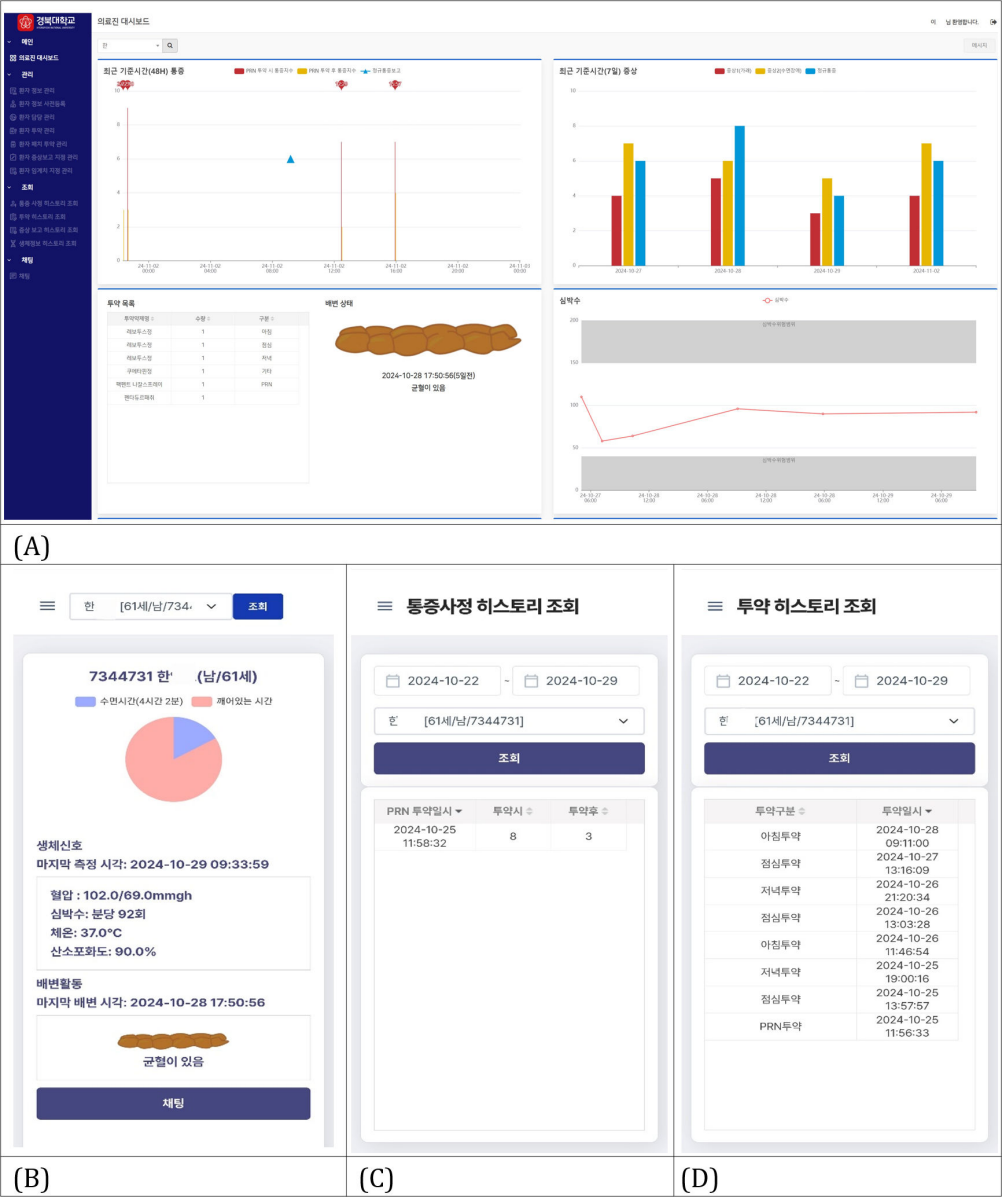
Web and mobile app interfaces for providers. (A) Main dashboard in the desktop view. (B) Main dashboard on the smartphone. (C) Pain history screen on the smartphone. (D) Medication history screen on the smartphone.

The provider-facing web interface received real-time data from patients, including symptoms, biosignal signals, and medication records. Providers could review trends and set personalized threshold–based alerts to facilitate timely interventions. This interface enabled the care team to assess medication adherence—particularly for PRN usage—and evaluate pain control through linked pre- and postdose data. Customizable alerts and a patient-specific dashboard allowed rapid clinical decision-making in home-based palliative care (Figure 4).

Although not directly used for clinical care, the administrative interface supported system management functions, such as account creation, patient registration, and alert configuration. It also enabled access management for providers and remote troubleshooting for system components (eg, connectivity, syncing errors). The admin panel served as the backbone for operational coordination and data integration across devices and users.

### User Demographics

Of the 7 patient-caregiver dyads, 5 successfully utilized the Hospice@Home system for 3 weeks. The patients ranged in age from 53 to 93 years (mean age 73.4, SD 15.7) and included two women and three men. Four had a high school and below education, and 1 held a college degree. All patients were married and living with their spouses, except 1 who was widowed. All patients had terminal cancer, with illness durations from 4 months to 8 years; however, all had received hospice care for less than 2 weeks at the time of enrollment. Three patients had 1 or more comorbidities, including hypertension (n=3), diabetes (n=1), benign prostatic hypertrophy (n=1), and chronic obstructive pulmonary disease (n=1). Prevalent target symptoms were pain (n=4) and loss of appetite (n=3), followed by weakness, pruritus, constipation, dizziness, urinary disorder, edema, and sleep disturbance.

Caregivers (1 sister, 4 daughters) were aged 38-63 years old (mean age 47.6, SD 9.8). Four had college degrees. The average duration of daily caregiving was 9.1 hours (range: 2.5‐21 h). The visiting nurse was a hospice advanced practice nurse in her early 40s with over 15 years of clinical experience.

### Reliability of Biosignal Measurements From the Galaxy Watch 6

The biosignal ICC values were as follows: systolic BP: ICC=0.679, 95% CI 0.103-0.782, *P*=.007; diastolic BP: ICC=0.495, 95% CI –0.812 to 1.949, *P*=.09; PR: ICC=0.985, 95% CI 0.956-0.994, *P*<.001; and O_2_Sat: ICC=0.771, 95% CI 0.313-0.923, *P*=.005. Additionally, significant correlations were found across the 18 paired measurements (Pearson *r*=.921, *df*=16, *P*<.001). The minimum ICC of 0.75 is generally recommended for acceptable reliability in biosignal measurements [29]. Based on this criterion, PR and O_2_Sat readings from the Galaxy Watch 6 demonstrated acceptable reliability, while BP measurements fell below the recommended threshold. Notably, in 1 case involving a patient with arrhythmia, a significant discrepancy was observed between the reference device (Omron HEM-7121J: BP: 129/50; PR: 43) and the Galaxy Watch 6 (BP: 89/54; PR: 65).

### User Engagement and Compliance With the System

**Figure 5.**
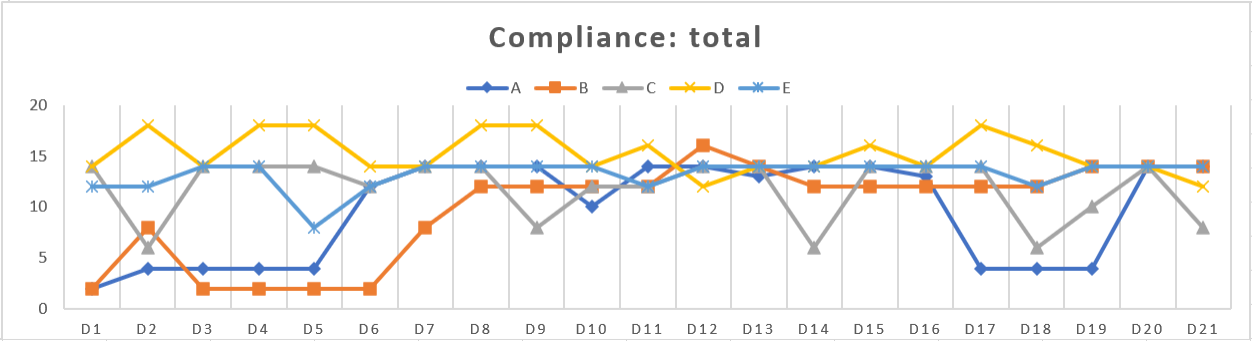
Patient compliance with the Hospice@Home system.

User compliance varied by participants’ familiarity with the technology and their health status, without a consistent pattern across users (Figure 5). Daily compliance scores were calculated based on 4 monitoring categories—vital sign measurements, symptom reporting, PRN pain score reporting, and scheduled medication adherence—using a predefined scoring system, with a possible range of 0-18 points per day. In this study, observed daily scores ranged from 2 to 18 across participants.

Participant A initially showed low compliance with vital sign monitoring due to a poorly fitting Galaxy Watch 6 sensor strap. Replacing it with a metal strap improved sensor contact and biosignal data collection. However, during days 17‐19, overall compliance, including vital sign measurements, symptom reporting, PRN medication logging, and scheduled medication adherence, temporarily declined due to an emergency room visit in response to worsening symptoms. Compliance levels recovered after this period. Participant B was unable to record biosignal data in the early phase of testing due to delirium and unstable vital signs. Following clinical stabilization, the participant achieved consistent and stable data entry across all monitoring categories, including vital signs, symptom reports, PRN medication use, and scheduled medication. Participant C maintained generally high engagement, with occasional lapses in scheduled medication adherence and vital sign reporting, particularly on days 9 and 19. Despite these fluctuations, this participant sustained near-daily use of core functions throughout the testing period. Participant E, the oldest participant, demonstrated stable and consistent compliance in all 4 areas throughout the testing period. Her daughter assisted with monitoring and reporting, contributing to steady performance in vital sign measurements, daily symptom logging, PRN medication, and scheduled medication intake.

### User Experience and Satisfaction

Overall satisfaction scores averaged 3.3 (SD 0.5) among patients and 4.0 (SD 0.7) among caregivers. Ease of use received comparable scores from patients (mean 3.3, SD 1.0) and caregivers (mean 3.2, SD 1.3). Perceived helpfulness was rated, with means of 3.5 (SD 1.0) for patients and 3.6 (SD 0.5) for caregivers. Caregivers were more likely to recommend the system (mean 3.8, SD 0.4) compared to patients (mean 3.3, SD 0.5). Communication improvement was rated at 3.3 (SD 1.0) by patients and 3.6 (SD 0.9) by caregivers.

Qualitative data revealed both positive experiences and areas requiring refinement across patients, caregivers, and health care providers. Patients generally found the system useful for structured care and symptom monitoring. Some appreciated the ability to track biosignal data through the wearable device. However, others reported difficulties in using the device consistently. For example, 1 participant stated, “I feel so fatigued that the watch feels heavy, and it’s difficult to measure four times daily” (participant A). Patients also expressed a preference for direct phone calls over the in-app chat feature, suggesting that synchronous communication was more reassuring in the home hospice context.

Caregivers reported several benefits, particularly valuing the system’s role in enhancing patient safety and enabling timely decision-making at home. For instance, 1 caregiver shared, “My father had dizziness, so I measured his oxygen saturation with the watch and discovered his oxygen level was low. I was relieved when his dizziness improved after applying oxygen immediately” (participant D’s daughter). Another caregiver emphasized the system’s impact on medication management. “Previously, my father would take medication whenever he felt he needed it, which worried me. I appreciated that he didn’t overuse his medicine since he didn’t access the doses stored in the medicine box” (participant E’s daughter). Nonetheless, some caregivers encountered challenges, particularly in supporting patients with cognitive or behavioral issues. One caregiver noted, “My mother has trouble sleeping and experiences delirium, so she doesn’t cooperate and keeps moving, which prevents the watch from taking measurements” (participant B’s daughter). Like patients, caregivers also favored phone calls over the system’s chat function, highlighting the need to improve communication features.

Health care providers, 1 physician and 2 home hospice nurses, highlighted the system’s clinical value. They noted that receiving objective information on patients’ conditions prior to visits facilitated more efficient care planning. The ability to monitor real-time medication adherence was also viewed as a major advantage. However, providers expressed concerns about the system’s lack of integration with the hospital’s electronic medical record (EMR) system. As a result, nurses were required to manually reenter patient data into the system during registration, leading to increased administrative burden.

## Discussion

### Principal Results

The adoption of DH technologies in HPC has steadily increased, enhancing information sharing, communication, and caregiver education [30]. This study developed Hospice@Home, an integrated system that combines patient monitoring, symptom and medication management, and prompt clinical response. By providing real-time data, the system supports clinical decision-making, improves communication, and complements face-to-face visits rather than replace them.

Although digital interventions can improve care coordination, they often impose a substantial data-entry burden on users, particularly those with advanced illness or limited digital literacy [31]. Hospice@Home sought to mitigate this barrier through individualized symptom targeting and automated medication tracking using an SMB. This design likely reduced redundant data entry and simplified routine use, which may explain the relatively high compliance observed among participating dyads. However, some patients still experienced data entry fatigue or cognitive difficulty in interacting with the wearable device, highlighting the continuing challenge of balancing data accuracy with usability in this population. While many mobile cancer-symptom management apps rely on fixed sets of standardized symptom items [32], Hospice@Home incorporated user-selected symptoms and PRN opioid monitoring, allowing more tailored and clinically meaningful data capture. This individualized approach enabled providers to assess medication efficacy in real time and to adjust care plans promptly. Nonetheless, personalization may also complicate data comparability across patients, which should be addressed in future system iterations and larger-scale validation studies.

Participants in the pilot study varied in age, health condition, and technological familiarity, which influenced their patterns of system use. Some initially expressed reluctance to engage with unfamiliar devices or reported fears of making errors. Nevertheless, the touch-based interface was generally perceived as intuitive and easy to learn, even among older adults. Notably, the oldest participant, who received assistance from her daughter, demonstrated consistently high compliance across all system features. This underscores the pivotal role of family caregivers in the adoption and sustained use of DH technologies—particularly for patients with terminal illnesses with significantly reduced functional capacity. For patients to remain at home until the end of life, technological solutions must be designed to alleviate, rather than add to, caregiver burden [33]. For home-based hospice care to remain sustainable, systems such as Hospice@Home must evolve toward greater automation, simplified feedback loops, and integration with existing care routines. The observed variation in user engagement highlights the need for adaptive designs that account for differences in digital literacy, functional status, and the level of caregiver involvement.

Wearable devices provide valuable objective data to complement subjective symptom reporting [34]. In this study, the Galaxy Watch 6 showed high ICCs for PR and O_₂_Sat, indicating acceptable reliability for physiological monitoring in home hospice care. However, blood pressure readings demonstrated lower reliability, falling below commonly applied thresholds for acceptable agreement. This was especially evident in a participant with arrhythmia, where discrepancies between wearable and manual measurements were substantial. Such inaccuracy may undermine clinical decision-making and pose safety risks if treatment adjustments rely on erroneous data, reflecting the variable measurement accuracy often reported for consumer-grade wearables in complex clinical conditions [35]. These findings suggest that wearable-derived blood pressure values should be interpreted cautiously in patients with terminal illnesses. Robust validation using diagnostic performance metrics—such as sensitivity, specificity, and predictive accuracy—will therefore be essential before clinical adoption. Although accuracy studies on wearable devices are accumulating, most have been conducted in healthy or mildly ill populations [34,35]. Empirical data remain limited for patients experiencing extreme physiological fluctuations, such as those in terminal illness, where unstable hemodynamics may further compromise measurement accuracy. In addition, although sleep duration was monitored using the Galaxy Watch 6, recorded sleep time often overestimated actual sleep reported by patients. This likely reflects the tendency of patients with terminal illnesses to spend extended periods lying down without truly sleeping. Frequent device removal during the night, especially among patients with advanced illness and reduced stamina, further limited continuous use, underscoring the need to adapt wearable technologies to the physical realities of terminal illness rather than assuming uninterrupted adherence. Such challenges, combined with the profound impact of sleep disturbance on quality of life in patients with terminal illnesses, highlight the need for alternative strategies to improve the accuracy of sleep monitoring.

User compliance varied depending on participants’ health status, comfort with technology, and the presence of caregiver support. Daily compliance scores across the 4 monitoring domains—vital signs, symptom reporting, PRN pain reporting, and scheduled medication adherence—ranged from 2 to 18 points (maximum possible: 18). Participants who appeared more comfortable using the technology or had consistent caregiver assistance tended to show more stable engagement, while temporary declines in compliance were observed during periods of clinical deterioration or device-related discomfort. Although the small sample size limits generalizability, these observations may be considered in relation to the technology acceptance model, which proposes that perceived ease of use and perceived usefulness influence an individual’s intention to adopt technology [36]. In particular, functional decline, older age, and limited technology literacy often necessitated caregiver mediation, suggesting that adoption in HPC settings is influenced not only by individual factors but also by relational and contextual variables. The variation in user engagement and feedback further highlights the importance of social support and user confidence in the successful adoption of DH systems in HPC settings. In this study, most data entry was performed by caregivers rather than patients, reflecting both the advanced illness of participants and the practical challenges of sustained self-reporting. This reliance underscores how caregiver involvement mitigates patient burden but may also introduce risks of data entry fatigue and intrusiveness, which should be considered in future system design.

Participants appreciated the system’s ability to consolidate symptom tracking, medication adherence, and vital sign data in a single interface. This integrated design may reduce cognitive burden and improve perceived control, both of which are critical for patients experiencing high symptom burden and uncertainty. The RFID tagging system allows patients and caregivers to directly report medication administration, ensuring accurate medication records. However, attaching an RFID tag after each dose, especially during nighttime medication, was perceived as inconvenient. To address this issue, an alternative solution could involve automatically transmitting medication information when the medication is dispensed from the SMB without RFID tagging. However, this approach may not always accurately reflect actual medication intake, as discrepancies could arise between dispensed and consumed medication. For example, medication could be dispensed from the SMB without the patient actually taking it, leading to inaccurate records. Therefore, this study prioritized the RFID tagging method to ensure medication record accuracy. This calls for future development of solutions that can achieve both accurate medication tracking and greater ease of use by integrating direct device to app data transfer. Additionally, the drawer-type SMB addressed issues related to limited hand strength, making it more accessible for patients with advanced illness and was preferred by patients with terminal illnesses and their families.

Although the app included an in-app chat feature, both patients and caregivers preferred phone calls, suggesting a mismatch between system design and users’ interpersonal expectations. While remote monitoring technologies have proven effective in chronic disease management, concerns remain about reducing interpersonal contact and increasing patient responsibility [37]. In the hospice context, the preference for phone calls likely reflects not only the need for real-time communication and timely clinical response but also the desire for human reassurance and emotional connection. For patients nearing the end of life, a compassionate and responsive human presence often provides greater comfort than remote monitoring data alone. This finding aligns with previous research indicating that after-hours telephone contact in hospice care primarily serves to provide reassurance in response to patients’ and caregivers’ anxiety and uncertainty [38]. To better reflect user preferences, communication features should be redesigned to facilitate more face-to-face-like interactions. Beyond improving communication, the successful implementation of DH technologies requires addressing broader systemic barriers, particularly those related to integration with existing health care infrastructure and minimizing disruption to clinical workflows [39]. This tension between automation and relational care highlights a key challenge in palliative care: maintaining interpersonal relationships while leveraging digital tools. Future system design and health care policy should encourage hybrid care models that combine automated monitoring with meaningful, human-centered interactions.

At the system level, clinician feedback identified the lack of integration with the hospital EMR system as a significant barrier to efficient workflow. The Hospice@Home system required manual re-entry of patient data into the EMR, resulting in duplicated tasks and increased administrative burden. While this was manageable during small-scale pilots, this could pose substantial challenges for routine clinical practice. Ensuring interoperability between home-based digital systems and institutional EMRs remains a global challenge due to stringent privacy regulations, firewalled hospital networks, and heterogeneous data standards [40]. Future development should therefore focus on establishing secure data exchange frameworks and standardized interoperability protocols that enable real-time integration while maintaining robust data protection and cybersecurity compliance. Integration with EMR systems will be essential to improve workflow efficiency, provide real-time access to monitoring data, and ensure continuity of care across home, emergency, and inpatient settings. To ensure smooth adoption, system development should involve clinicians and IT staff in codesign and proceed through stepwise pilot testing. For long-term success, system architectures must be designed with compatibility and interoperability in mind.

### Recommendations for Future Research

Improvements should focus on simplifying the medication recording process by replacing RFID tagging with a more user-friendly method integrated into the dispenser. Enhancing the reliability of vital sign monitoring, especially for patients with arrhythmia, hypotension, or poor peripheral circulation, is essential for safe and informed clinical decision-making. Increasing accessibility by making the mobile app easier to download and use can further boost engagement. Future system development should also include EMR integration to support scalability and routine clinical use.

### Limitations

This pilot study has several limitations. The small sample size, 5 patient-caregiver dyads, limits generalizability. Recruitment was difficult due to the frailty and fluctuating symptoms of home hospice patients. Moreover, the small number of screened dyads and reasons for nonparticipation may reflect potential selection bias, further limiting generalizability. The study was conducted in a single geographic and cultural context (South Korea), which may affect its applicability to more diverse populations or health care settings. System development relied on input from an SME team that consisted only of health care providers, without the inclusion of psychosocial team members, which may limit the breadth of perspectives incorporated. Usability was also assessed using a simplified, nonvalidated questionnaire developed for this pilot study. This approach facilitated feasibility in a vulnerable population but limits comparability with studies using standardized tools. While provider perspectives were incorporated during the system development phase through the SME team, the usability evaluation in this pilot study focused only on patients and caregivers and did not formally assess provider usability, which may limit the comprehensiveness of usability findings. In addition, the absence of validated outcome measures, including standardized usability scales and clinical endpoints, further restricts the interpretability and generalizability of the findings. Clinical outcomes such as long-term symptom control and caregiver burden were not assessed. Moreover, potential biases, such as volunteer bias, social desirability in satisfaction ratings, and short-term Hawthorne effects, may have influenced the findings. Finally, the system was not integrated with hospital EMRs, requiring the manual re-entry of patient information and increasing the administrative burden, which may have affected user satisfaction.

### Conclusions

This pilot study explored the feasibility and usability of Hospice@Home, a DH system designed to support HPC in South Korea. The system integrated wearable biosignal monitoring, PRN medication management through an SMB, and electronic symptom reporting to enhance communication and HPC coordination. Preliminary findings indicate that the system appeared to be usable and acceptable within a small sample of patients with terminal illnesses and caregivers, but these results should be interpreted cautiously given the homogeneous cohort, the very limited sample size, and the use of nonvalidated usability measures. The short duration of the testing also precludes any definitive conclusions about clinical effectiveness. These findings, therefore, emphasize the preliminary potential of the system. Future research should involve larger and more diverse samples, validated outcome measures, and longer follow-up periods to evaluate the system’s clinical impact, patient outcomes, and sustained engagement over time. Based on the key barriers identified in this pilot, immediate priorities for further development include streamlining data entry workflows, improving biosignal accuracy (particularly for blood pressure), and achieving EMR integration to reduce administrative burden and ensure continuity of care.

## Abbreviations

AHEAD: Approach to Human-centered, Evidence-driven Adaptive Design
BP: blood pressure
DH: digital health
EMR: electronic medical record
HPC: home-based palliative care
ICC: intraclass correlation coefficient
O_2_Sat: oxygen saturation
PCU: palliative care unit
PR: pulse rate
PRN: pro re nata
RFID: radio frequency identification
SMB: smart medication box
SME: subject matter expert
